# Special Issue “Cellular and Molecular Progression of Cardiovascular Diseases”

**DOI:** 10.3390/ijms27041872

**Published:** 2026-02-15

**Authors:** Andrea Borghini, Antonio Rizza, Alessandro Tonacci

**Affiliations:** 1 CNR Institute of Clinical Physiology, 56124 Pisa, Italy; alessandro.tonacci@cnr.it; 2 U.O.C. Cardiologia Diagnostica e Interventistica, Fondazione Toscana Gabriele Monasterio, 54100 Massa, Italy; antonio.rizza@ftgm.it

Cardiovascular disease (CVD) currently ranks as the leading cause of global mortality and morbidity. In 2022, approximately 20 million people lost their lives due to CVDs, accounting for about 32% of all global fatalities [1,2]. 

CVDs include a range of disorders affecting the heart and blood vessels, including coronary heart disease, cerebrovascular disease, peripheral arterial disease, congenital heart defects, rheumatic heart disease, and deep vein thrombosis and pulmonary embolism. Hypertension and diabetes mellitus are important risk factors for CVD and are prevalent comorbidities [3]. This double-risk situation rises from shared risk factors and coinciding pathological pathways—such as inflammation, oxidative stress, and insulin resistance—that substantially increase the likelihood of CVD complications [4].

Despite extensive research, gaining novel insights into the pathophysiology and molecular mechanisms of CVDs through both basic and clinical research remains crucial. This effort is essential to identify new diagnostic and prognostic markers and to develop therapeutic strategies that can enhance patient management and outcomes [5,6]. 

The integration of “omic technologies” has transformed cardiovascular research by enabling the high-throughput investigation of biological systems at different molecular levels [7]. These tools are advancing precision medicine by uncovering the molecular mechanisms underlying CVD and supporting a bottom-up approach that focuses on molecular components initially and then integrates findings into larger systems [7].

Additionally, artificial intelligence stands out as a powerful model capable of recognising complex patterns of CVDs within large-scale molecular and clinical data. Its potential to improve risk prediction is considerable, allowing for the identification of markers for more accurate diagnoses, the prediction of treatment outcomes, and the development of innovative therapies for more precise and tailored approaches [8]. The aim of this Special Issue was to cover the most recent advances in the molecular and cellular mechanisms underlying CVDs, as well as the utility of artificial intelligence in interpreting and integrating clinical and molecular data.

Due to the contribution of international research groups, in addition to this editorial, this Special Issue includes 12 papers—comprising six original articles and six reviews—covering a wide range of topics; Figure 1 summarises these papers.

In the first contribution by Nyulas et al., the authors reviewed the cardioprotective effects of various phytotherapy agents (e.g., garlic, aloe vera, green tea, ginkgo biloba, berberine) in depth, highlighting their potential role as beneficial options for the management of hypertension and related complications. Emerging evidence indicates that traditional medicine can be integrated into modern personalised treatment plans as a complementary approach alongside standard pharmacological therapies. This integration aims to optimise treatment effectiveness, while reducing the risk of harmful interactions with current medications [contribution 1]. 

Three sequential reviews, conducted temporally, address the topics of atherosclerosis and myocardial infarction, each examining different aspects [contribution 2–4]. Specifically, Daher and colleagues focused on the effects of radiotherapy on coronary artery disease, a topic of great interest. They explored the underlying cellular (endothelial dysfunction, inflammation, fibrosis) and molecular mechanisms (oxidative stress, DNA damage, telomere erosion, mitochondrial dysfunction) and potential strategies to prevent radiotherapy-induced effects. Interestingly, among several strategies reviewed for preventing cardiovascular complications in cancer patients, progress in radiotherapy—particularly the novel FLASH effect—shows promise in reducing these complications [contribution 2]. This radiation modality specifically targets tumour tissues with very high-intensity beams while simultaneously protecting neighbouring normal tissues, sparing them from the toxic effects of radiation [9].

In a review by Tonch-Cerbu et al., the authors investigated the current understanding of the mechanistic, diagnostic, and therapeutic connections between gut microbiota and atherosclerosis. They consolidated the evidence supporting the gut microbiota as a crucial factor in the development of atherosclerosis, with particular emphasis on microbial products such as trimethylamine N-oxide, lipopolysaccharides, and short-chain fatty acids and their roles in this process [contribution 3].

The fourth contribution offers a comprehensive and well-articulated overview of the roles of neutrophils and monocytes in the pathophysiology of myocardial infarction. It explored the mechanisms of intercellular communication—particularly through neutrophil-derived secreted factors—that modulate immune system, thereby influencing post-MI inflammation and tissue repair. Additionally, it highlighted potential therapies aimed at modulating inflammation and the cross-talk between neutrophils and macrophages [contribution 4].

In early-stage type 2 diabetes mellitus, patients often exhibit autonomic cardiovascular dysfunction characterised by reduced parasympathetic activity in the heart, leading to an increased risk of arrhythmias and sudden cardiac death. The regulation of cardiac parasympathetic tone involves neural circuits at different levels, including afferent, central, and efferent pathways. Specifically, efferent control is mediated by preganglionic neurons in the vagus nerve that activate postganglionic neurons within intracardiac ganglia, which release neurotransmitters to exert local cardiac regulation. Structural and functional changes in these postganglionic neurons impair parasympathetic control, promoting arrhythmias and sudden death. Understanding the remodelling of parasympathetic postganglionic neurons and the molecular mechanisms underlying the reduction in parasympathetic activity could provide insights into therapies aimed at restoring cardiac parasympathetic tone, potentially reducing arrhythmias and improving the survival and quality of life in patients with diabetes. This complex topic is thoroughly addressed in the fifth review [contribution 5].

In the final review in this Special Issue, Fontanelli and colleagues discussed how human-induced pluripotent stem cell-derived endothelial cells (iPSC-ECs) can serve as personalised experimental platforms in cardiovascular and cerebrovascular diseases. Interestingly, the authors underscored the potential of this model system to develop into clinical decision-support to advance precision medicine. One approach is endothelial drug profiling, where patient-specific iPSC-ECs are exposed to stressors such as inflammatory cytokines, shear stress, and hypoxia, followed by testing therapies to identify the most effective treatment. Another approach is endothelial risk profiling, which employs standardised tests to assess endothelial reserve and vulnerability, thereby helping to predict disease risk and potential complications. 

In the context of environmental cardiology, they emphasised that the use of iPSC-ECs represents a groundbreaking advancement in the study of environmental toxins’ effects on human health. By leveraging these innovative models, scientists can gain profound insights into the complex interplay between genetic predispositions and environmental exposures, particularly in how such interactions can accelerate the development of cardiovascular diseases. This approach not only facilitates the early detection of toxic effects and underlying disease mechanisms more rapidly than a traditional epidemiological approach but also accelerates the discovery of biomarkers and therapeutic targets, thereby advancing predictive and personalised medicine [contribution 6].

The original articles address different aspects of CVD [contributions 7–12]. In contribution seven, Koenig’s group aimed to determine the expression and function of Ankyrin-R, a protein responsible for directing ion channels and transporters to the cell membrane, in the heart. Since the typical form of Ankyrin-R had not been previously studied in cardiac fibroblasts, the authors focused on assessing its presence in these cells. They found that a smaller isoform of Ankyrin-R is the most prevalent in cardiomyocytes, whereas the canonical Ankyrin-R is the dominant form in cardiac fibroblasts, which displays perinuclear and cytoplasmic expression and co-localises with the Golgi apparatus. Moreover, by using conditional knockout mice, these results show that the deletion of Ankyrin-R in activated fibroblasts modifies the composition of collagen fibres and impairs fibroblast function [contribution 7].

The eighth contribution focused on comparing the impacts of the primary volatile anaesthetics on the balance between pro-oxidant and antioxidant activity during endovascular aneurysm repair, the main surgical method for treating aortic aneurysms. During the procedure, inflammation and ischemia–reperfusion injury contribute to heightened perioperative oxidative stress, which is linked to increased postoperative complications. Utilising a comprehensive measure of oxidative stress known as the OXY-SCORE—derived by integrating various plasma biomarkers of oxidative damage and antioxidant defences—the authors demonstrated, for the first time, that desflurane exerts a favourable effect on oxidative stress levels during this surgical intervention [contribution 8]. 

In a prospective study, a research team from Italy conducted an in-depth investigation into inflammatory processes associated with chronic venous disease, emphasising the impairment of the endothelial glycocalyx and exploring the therapeutic potential of mesoglyca, a glycosaminoglycan-derived medication, on both local and systemic inflammation. Their study revealed that patients with varicose veins exhibited significantly higher levels of inflammatory and endothelial injury markers, including vascular Cell Adhesion Molecule-1, Matrix Metalloproteinases 2 and 9, Syndecan-1, Syndecan-4, and Interleukin-6, in the serum obtained from the affected veins compared to the veins in systemic circulation within the same individuals. Notably, the researchers also suggested that endothelial and glycocalyx damage might be at least partially reversible, as evidenced by improvements following a short-term course of glycosaminoglycan-based therapy [contribution 9].

In another pioneering study, Dong et al. showcased the significant potential of combining in vitro and in silico methods to discover therapeutic targets for cardiomyopathies. They present the first integration of a mathematical electrophysiological model, capturing fibroblast–cardiomyocyte interactions, with experimental engineered heart tissue research, aiming to identify and modulate the ion channels that control these interactions. Their findings underscore the critical importance of detailed ionic current modelling in cardiomyocytes for accurately predicting fibroblast–cardiomyocyte dynamics in engineered heart tissues [contribution 10].

Starting from the existing knowledge that oxidative stress, inflammation, and altered levels of miRNAs are involved in the pathological process of vascular damage leading to complications in diabetes, Duisenbeck et al. hypothesised that analysing selected miRNAs, combined with markers of oxidative stress and inflammation, may help identify the risk factors associated with vascular complications, including macrovascular events. To achieve this goal, the authors employed a descriptive cross-sectional design involving three groups of participants: 30 controls, 34 patients with diabetes, and 22 patients with diabetes and vascular complications. Overall, this study identified two models with high predictive value for the presence of vascular complications: (1) glycated haemoglobin, creatinine, total cholesterol, lipid peroxidation, glutathione peroxidase, superoxide dismutase, miRNA-126, and miRNA-484; and (2) glycated haemoglobin, creatinine, total cholesterol, Interleukin-6, lipoprotein oxidase, miRNA-126, and miRNA-484 [contribution 11].

The final original research article elucidated possible variations in cardiac performance, arrhythmogenicity, and protein levels after administering either one or three intracoronary cardiosphere-derived cells in a pig model of severe heart damage caused by myocardial infarction. The research aimed to evaluate the differences in safety and effectiveness and to identify potential molecular indicators of therapeutic success. Repeated cardiosphere-derived cell administration elicited potentially significant proteomic adaptations, particularly in pathways related to post-infarction repair. However, these molecular alterations did not translate into clear improvements in cardiac function. This disparity suggests that the incremental benefits observed at the proteomic level are insufficient to induce changes in cardiac performance, thereby limiting the direct translational potential of this therapeutic scheme [contribution 12].

Although focused on different topics, the 12 papers included in this Special Issue highlight the need for further comprehensive research into the pathophysiology of CVDs. The findings presented are valuable clues for the diagnosis of CVD and can serve as a foundation for developing effective therapies and designing novel drugs. 

## Figures and Tables

**Figure 1:**
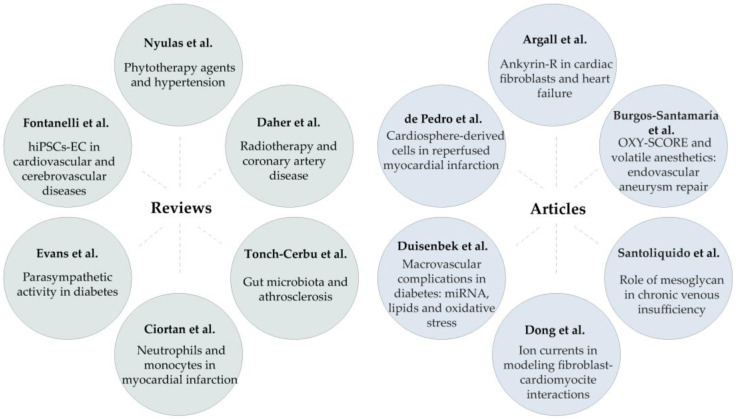
Schematic representation of the contributions published in the Special Issue “Cellular and Molecular Progression of Cardiovascular Diseases”, including a broad spectrum of topics.
